# Acute salivary gland hypofunction in the duct ligation model in the absence of inflammation

**DOI:** 10.1111/j.1601-0825.2007.01413.x

**Published:** 2008-09

**Authors:** PN Correia, GH Carpenter, SM Osailan, KL Paterson, GB Proctor

**Affiliations:** Salivary Research Unit, King's College London Dental Institute at Guy's, King's College and St Thomas’ HospitalsLondon, UK

**Keywords:** atrophy, dexamethasone, hypofunction, inflammation, saliva, salivary gland

## Abstract

**Objective:**

The commonly associated aetiology of salivary gland inflammation and salivary hypofunction has led to the widely held belief that inflammation causes salivary gland hypofunction. Indeed, our own recent study seemed to support this contention. Here, we tested the hypothesis that, in an acute duct ligation model, eliminating inflammation the submandibular gland would recover normal function.

**Materials and methods:**

Ligation of the rat submandibular gland excretory duct for 24 h was used to induce inflammation and salivary gland hypofunction. A group of duct ligated rats was compared with a second group given dexamethasone, on the day of duct ligation. Twenty-four hours later salivary gland function was assessed and salivary glands were collected.

**Results:**

Histology and myeloperoxidase activity assay revealed a profound decrease in inflammatory cell infiltration of ligated glands from rats given dexamethasone, compared with ligated glands in the absence of dexamethasone. Salivary flow rate evoked by methacholine was decreased (*P* < 0.01) by approximately 56% (ligated *vs* control, 79 ± 9 *μ*l min^−1^ g^−1^*vs* 177 ± 11 *μ*l min^−1^ g^−1^) and salivary flow from ligated dexamethasone-treated and ligated glands was similar.

**Conclusion:**

Despite eliminating the inflammatory reaction in the ligated gland, salivary hypofunction was not reversed, suggesting that other mechanisms must be at work in the ligation-induced salivary hypofunction.

## Introduction

The majority of salivary gland pathology is inflammatory and most salivary gland inflammatory diseases have in common an associated salivary hypofunction (Van Den Akker and Busemann-Sokole, 1983; Beale and Madani, 2006; Dawson *et al*, 2006). Through sialometry, salivary flow rate can be determined, confirming salivary gland insult (Mandel and Baurmash, 1980). Impaired salivary glands also show infiltrating inflammatory cells, suggesting that the onset of tissue injury requires the activation of infiltrating cells (Humphreys-Beher and Peck, 1999; Fox and Stern, 2002; Kimura-Shimmyo *et al*, 2002). Current evidence suggests that inhibition of salivary secretion may precede and continue along with tissue destruction (Humphreys-Beher and Peck, 1999; Fox and Stern, 2002). A transient inflammation of salivary glands, due to ductal ligation, was reported to trigger an autoimmune disease in some mouse stains. Recently, we have demonstrated that salivary hyposecretion, associated with 24 h duct obstruction, was reversed following 3 days of deligation and that isolated acinar cell preparations, from ligated glands, showed a normal elevation of intracellular calcium in response to cholinergic stimulation. These findings suggest that salivary hypofunction is reversed when the cells are separated from the surrounding inflammatory infiltrate (Carpenter *et al*, 2007). Nonetheless, the contribution of infiltrating cells to salivary gland hypofunction is still not fully elucidated.

Ligation of the rat submandibular gland excretory duct has contributed to the understanding of the pathology of duct obstruction (Cummins *et al*, 1994), which ultimately leads to progressive atrophy of the affected gland (Harrison *et al*, 1997). The aetiology of duct obstruction is multiple, but by far the most frequent cause is salivary calculi formation. Sialolithiasis is the commonest cause of salivary gland disease presented to the general dental practitioner, most often affecting the submandibular gland (Lustmann *et al*, 1990), and also seen in the parotid as chronic obstructive parotitis (Wang *et al*, 1992; Qi *et al*, 2005). Although less frequent, the main glandular duct can become compressed by the growth of a tumour (e.g. salivary duct carcinoma) (Giger *et al*, 2005) or become obliterated because of salivary duct strictures (Drage *et al*, 2002).

Following long-term duct ligation, there is disorganization of the gland parenchyma, dilation of ducts and progressive atrophy. Infiltrates of neutrophils occur in the early stage of obstruction (1–18 h) followed by a monocyte invasion evident by 24 h (Standish and Shafer, 1957; Tamarin, 1979; Norberg *et al*, 1988). At this early stage there is a decrease of saliva secretion in response to all types of secretagogues (Carpenter *et al*, 2007). There are several signalling pathways involved in the control of secretion that can be affected by proinflammatory cytokines (IL-*β*_1_, IL-6 TNF-*α*) and prostaglandins (Yu, 1986; Tanda *et al*, 1998; Yamakawa *et al*, 2000). Nitric oxide can have a dual effect in salivary gland secretion: it can mediate cytokine-induced salivary dysfunction, and it can also participate in the physiological neural regulation (Rettori *et al*, 2000; Rosignoli *et al*, 2001). From 48 h of duct ligation onwards, glandular atrophy becomes obvious with secretory cell degranulation (Osailan *et al*, 2006b), which develops to a progressive decrease in secretory function and gland weight (Martinez *et al*, 1982).

Dexamethasone has been used in different studies, whenever it is desirable to limit inflammation and prevent tissue destruction (Jick *et al*, 1965; Masferrer *et al*, 1990; Adesanya *et al*, 1996; Yang *et al*, 1998; Perretti *et al*, 2002). This glucocorticoid inhibits the generation of eicosanoids and superoxide anion and is also a potent suppressor of iNOS expression (Robbins *et al*, 1994). In the present study, the duct ligation model was used to induce acute inflammation of the submandibular gland and dexamethasone was used as an anti-inflammatory to further test the hypothesis that early salivary hypofunction associated with duct ligation is caused by inflammation.

## Materials and methods

### Rat submandibular duct ligation – surgical procedure

Young adult male Wistar strain rats (Harlan Labs Ltd, Loughborough, UK), 192–281 g, were housed under standard conditions (12 h light/dark cycle at 22–25°C) with a chow pellet diet and water *ad libitum*. Animal work was performed according to Home Office regulations (guidance on the operation on animals was from the Scientific Procedures Act 1986). The animals were weighed and given Ketamine (75 mg kg^−1^) and Xylazine (15 mg kg^−1^) i.p., as an equal mixture of each drug. After the rats were anaesthetized, an intra-oral approach was taken to dissect the main excretory duct of the right submandibular gland, through a small incision in the floor of the mouth, to avoid the chorda-lingual nerve (Osailan *et al*, 2006b). The duct was ligated 5 mm posterior to the ductal orifice with a metal micro clip (SLS™-CLIP, Vitalitec, Domalain, France) and the incision was closed with 8/0 Ethilon suture (ETHICON Inc., Johnson & Johnson Health Care Systems, Piscataway, NJ, USA). In seven out of 14 rats, dexamethasone i.m. (5.5 mg kg^−1^) was administered before recovery from anaesthesia. Aseptic conditions were used throughout the surgical procedure of duct ligation to reduce the risk of infection. In all animals, the contralateral, unligated left submandibular gland was used as a control.

### Saliva collection

Gland stimulation was performed 24 h after the duct ligation. Rats were weighed and anaesthetized with pentobarbitone (50 mg kg^−1^) i.p. and secured in the supine position on a heated table. A cannula was introduced into the femoral vein. Chloralose (80 mg kg^−1^) was delivered i.v. via the femoral vein to maintain long-term anaesthesia and additional pentobarbitone was given, if necessary. The trachea was cannulated so that clear airways would be provided during cannulation (as methacholine induces secretion in all body fluids, including mucus secretion in the lungs, which can cause respiratory distress). Body temperature was recorded by rectal thermometer and maintained at 38°C. Saline was given i.p. to maintain fluid levels, if required.

For collection of submandibular saliva, the ducts of the submandibular glands were exposed, from extra-oral approach, and cannulated proximal to the gland, with polyethylene cannulae (Osailan *et al*, 2006b). The right submandibular cannula was inserted posterior to the ligation. Salivation was stimulated with i.v. drugs, as described below, and saliva samples were collected into preweighed tubes, weighed, kept on ice and subsequently stored at −70°C.

A methacholine stock solution of 14.4 mg ml^−1^ (0.07 M) was diluted in saline to 144 *μ*g ml^−1^ (0.74 mM). It was delivered i.v. with a syringe pump rate adjusted to 80 *μ*l kg^−1^ body weight, such that methacholine was delivered at 12 *μ*g min^−1^ kg^−1^. This drug is a cholinergic agonist and induces saliva flow on binding to muscarinic receptors (Osailan *et al*, 2006b). Saliva was collected from both ducts for 2 min. These saliva samples contained accumulated levels of salivary protein, following anaesthesia, and were collected to check that the system was working, i.e. that saliva was stimulated and the cannulae were not leaking (Proctor *et al*, 2003a). To distinguish between different stimulations, a saline bolus injection was delivered i.v. between each stimulation. All subsequent saliva samples were collected for 5 min and amounts determined gravimetrically (Anderson *et al*, 1995). Isoprenaline, a *β*-adrenergic receptor agonist, strongly stimulates protein secretion from submandibular acinar cells (Proctor *et al*, 2003b). Isoprenaline 1 mg ml^−1^ (4 mM) stock was diluted to 5 *μ*g ml^−1^ (0.02 mM) and administered at a rate of 0.4 *μ*g min^−1^ kg^−1^. As such stimulation produces a slow flow of saliva, to allow a proper measurement of protein secretion, a high dose of methacholine (144 *μ*g ml^−1^) was added to isoprenaline. Immediately after the end of the collection period, each submandibular gland was removed, separated from the sublingual gland and weighed. The animals were killed with an overdose of pentobarbitone.

### Salivary glands sample preparation

After removal, each submandibular gland was divided into pieces. For biochemical analyses, tissue pieces were immediately frozen in liquid nitrogen. For morphology–immunohistochemical studies, tissues were placed in OCT (embedding medium, Raymond A Lamb Ltd., East Sussex, England) then frozen in isopentane and cooled in liquid nitrogen. For conventional histochemistry, tissues were fixed in formol sucrose (4% formaldehyde, 7.5% sucrose, 0.08 M cacodylate buffer pH 7.2).

### Histochemistry

Formol sucrose fixed tissue was processed to paraffin embedding and 5 *μ*m paraffin sections were cut, mounted on slides and stained with Ehrlich haematoxylin and 1% eosin (H&E). Alcian blue/periodic acid Schiff's stains (AB/PAS) were used for the demonstration of acinar and ductal secretory granules and *p*-dimethylaminobenzoaldehyde (DMAB) was used for granular convoluted ductal cell secretory granules.

### Morphometric analysis

An estimate of acinar areas was performed using Leica TCS SP2 software, version 2.1 (Leica Microsystems Heidelberg GmbH, Germany). From ligated, dexamethasone-treated ligated glands and respective contralateral control glands, 20 acini per gland were randomly selected and the mean area (*μ*m^2^) calculated.

### Total salivary protein assay

Total salivary protein was assayed spectrophotometrically (Abs 215 nm). Samples were diluted 1/100 with double distilled water and absorbance measured at 215 nm using a spectrophotometer (Ultrospec 4050, Biochrom, Cambridge, UK). Double distilled water was used as a blank and bovine serum albumin (BSA; Sigma, Poole, UK) was used to construct a standard curve.

### Total glandular protein assay

The Bicinchoninic Acid (BCA) protein assay kit (Pierce Biotechnology, Rockford, IL, USA) was used in a microplate. Submandibular gland homogenates were diluted (1/50) with water and then loaded 25 *μ*l in the wells. Subsequently, the BCA working reagent was added and the plates were incubated for 45 min at 37°C and afterwards allowed to cool down to room temperature. The sample reaction was read at 540 nm. A standard curve was created using BSA, to determine the protein concentration of each sample.

### Glandular peroxidase assay

Peroxidase was assayed enzymatically using the substrate 2,7-dichlorofluorescein diacetate (LDADCF; Molecular Probes Inc., Eugene, OR, USA) as described by Proctor and Chan (1994). LDADCF was activated to dichlorofluorescein (LDCF) with 1 M sodium hydroxide and then neutralized with 67 mM phosphate buffer (pH 6.0). Glandular homogenates, 4% (w/v) in phosphate containing buffer (pH 6), were centrifuged at 10 000 ***g*** for 10 min. The supernatant was removed and diluted 1/200 in phosphate buffer for assay. The samples were loaded on to a 96-well plate (Iwaki Co., Tokyo, Japan) and placed in a bath at 37°C. Hydrogen peroxide, phosphate buffer containing potassium thiocyanate and LDCF were added sequentially. The substrate reaction was stopped after 4 min by 1 M sodium hydroxide. Fluorescence of the product dichlorofluorescein (DCF) was read in a fluorimeter (PerkinElmer Life and Analytical Sciences, Milan, Italy) set at 485 nm excitation and 538 nm emission. DCF (Sigma Chemicals Ltd, Perth, Western Australia) was used as a standard by diluting 10 mM DCF stock to a 2 *μ*M solution (Proctor and Chan, 1994).

### Myeloperoxidase assay

Following centrifugation, the tissue pellets from glandular homogenates prepared as above, were resuspended in phosphate buffer containing 13.7 mM hexadecyltrimethylammonium bromide (Sigma Chemicals Ltd.) and 10 mM EDTA. The samples were diluted and loaded on to a 96-well plate and placed in a bath at 37°C. Hydrogen peroxide, phosphate buffer and tetramethyl benzidine (TMB) were added sequentially. The substrate reaction was stopped after 4 min by sulphuric acid. The absorbance of the sample reaction was read at 460 nm. Different dilutions of human myeloperoxidase enzyme of known concentration were used to obtain the standard curve (Sigma Chemicals Ltd.). Myeloperoxidase activity was expressed as mU per gram tissue. Dapsone (diaminodiphenylsulfone, Sigma Chemicals Ltd.), a selective inhibitor of peroxidases, other than myeloperoxidase, was used to confirm the specificity of the assay (Thomas *et al*, 1994).

### Direct potentiometry

The concentration of chloride ions in the saliva samples was assayed using an ion-selective electrode (ISE) in combination with a potassium nitrate reference electrode (ELIT Ion Analyser, Nico 2000 Ltd, Harrow, UK), eight channel ion analyser and computer interface software. Before use, the ISE was allowed to stand in a chloride solution of 1000 mg l^−1^ for 5 min or until the milliVolt (mV) output reached a stable reading. The ISE was calibrated by measuring two standard chloride solutions of 1 and 10 mg l^−1^, within the concentration range expected for the samples, and a straight line of mV *vs* concentration plotted to provide the calibration data. Between each measurement/sample, electrodes were washed with de-ionized water and dried to avoid cross contamination. The diluted saliva samples (1/100) were measured in the same way as the standards and the results displayed as mg l^−1^. To obtain a high precision, every 10 samples, recalibration was performed.

### Statistical analysis

Data were analysed using one-way ANOVA and paired Student's *t* test when comparing contralateral glands from the same group and unpaired Student's *t* test for comparisons between glands in dexamethasone-treated and untreated series. Results are expressed as means ± s.e.m. and *P* < 0.05 was considered significant.

## Results

### Submandibular tissue

**Figure 1:**
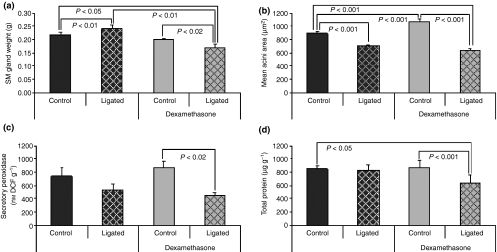
Effects of 24-h duct ligation with and without the presence of dexamethasone on submandibular gland weight and acinar cell size, stored acinar cell peroxidase and glandular protein content. Results were compared using paired and unpaired Student's *t* test and *P* < 0.05 considered significant. (**a**) Mean submandibular gland weight of 24-h ligated gland is increased comparing with control glands (*n* = 7). In contrast, dexamethasone-treated ligated glands are smaller than respective contralateral control (*n* = 7) and control and ligated untreated submandibular glands. (**b**) The mean area of acini from 24-h duct ligated glands and dexamethasone-treated ligated glands shows a reduction in the size of acini in the ligated glands (*n* = 100), and dexamethasone-treated ligated glands (*n* = 100), when compared with contralateral controls, (*n* = 100). Acinar size of control dexamethasone-treated glands was bigger than acini of control glands (unpaired Student's *t* test). (**c**) Secretory peroxidase activity from submandibular gland homogenates. In the dexamethasone-treated ligated group, after 24 h, the activity of peroxidase was significantly decreased, *n* = 5. In the 24-h duct ligation group, the difference was not significant, *n* = 6. (**d**) Total protein from submandibular gland homogenates. In the dexamethasone-treated ligated glands (*n* = 7), total protein content decreased compared with control dexamethasone-treated and untreated glands

After 24 h of duct ligation there was a significant increase (*P* < 0.01, *n* = 7) in the submandibular gland weight comparing with the contralateral gland. The mean weight of ligated submandibular gland in dexamethasone-treated rats was decreased by 20% (*P* < 0.02, *n* = 7) compared with the contralateral control submandibular gland (Figure 1a). There was no difference in the rat body weight between the dexamethasone treated and untreated groups (data not shown).

**Figure 2:**
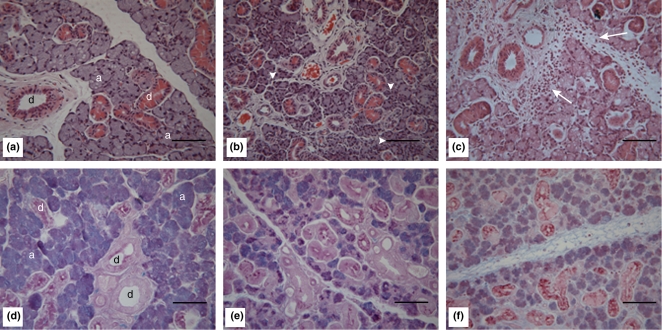
Morphology of stimulated submandibular gland. In the top row, H&E staining for general morphology and in the lower row, AB/PAS reagent staining showing salivary glycoprotein containing secretory granules. (**a**) and (**d**) Dexamethasone-treated control submandibular gland. (**b**) and (**e**) Dexamethasone-treated ligated submandibular gland. (**c**) and (**f**) Ligated submandibular gland. (**a**) and (**d**) Illustrate a normal submandibular gland histological architecture with abundant closely packed acini (**a**) and large spaced, different sized ducts (**d**). (**b**) Contains a condensed stroma with some atrophic acini (arrowheads) and shows a marked reduction in inflammatory cells compared with (**c**); arrows on (**c**) indicate inflammatory cells. (**e**) and (**f**) Contain fewer acinar cell secretory granules than (**d**). All micrographs are ×125 magnification. Scale bars = 20 *μ*m

As seen in Figure 2c, the H&E stained ligated gland shows a moderate inflammatory infiltrate and an increased extracellular space. The infiltrate was composed of neutrophils and macrophages and mainly dispersed in the intralobular and interlobular connective tissue. In the dexamethasone-treated ligated submandibular gland there was no infiltrating inflammatory cells and a marked reduction in the interstitial oedema compared with ligated submandibular gland without dexamethasone (Figure 2b,c). Acinar cells looked shrunken and the gland showed a more compact appearance in the dexamethasone-treated ligated gland (Figure 2b) when compared with the contralateral control gland (Figure 2a). Comparison of control glands showed that dexamethasone treatment *per se* did not cause obvious changes in glandular morphology (figure not shown).

Staining of acinar and ductal secretory material with AB/PAS showed that ligated and dexamethasone-treated ligated submandibular glands (Figure 2f,e), contained fewer secretory granules which were more localized in the apical pole, compared with respective control submandibular gland (Figure 2d). The DMAB staining, specific for stored tissue kallikreins of the granular convoluted ducts (Shori *et al*, 1997) did not show a substantial difference between ligated *vs* control glands and dexamethasone-treated ligated *vs* contralateral control glands (data not shown).

Quantitative morphological analysis indicated that the area of acini from ligated glands was significantly reduced, in both ligated (*n* = 6, *P* < 0.001) and dexamethasone-treated ligated animals (*n* = 5, *P* < 0.001), compared with their respective control glands (Figure 1b). Mean acini area from control dexamethasone-treated glands was higher than control glands, (*P* < 0.001, unpaired Student's *t* test).

**Figure 3:**
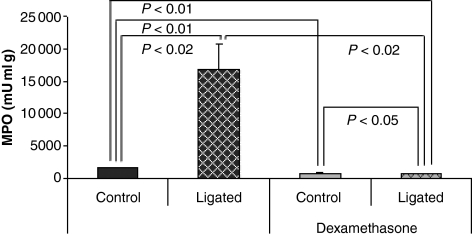
Effect of 24-h duct ligation with and without the presence of dexamethasone on myeloperoxidase activity. Myeloperoxidase activity after 24-h duct ligation is greatly increased, compared with control, *n* = 6. Dexamethasone dramatically reduced myeloperoxidase activity in duct ligated glands, *n* = 5. Myeloperoxidase was decreased in the control dexamethasone-treated group compared with the control group (unpaired Student's *t* test)

Duct ligation alone led roughly to a 10-fold increase in the myeloperoxidase activity (*n* = 6, *P* < 0.02), compared with the contralateral control gland (Figure 3). Dexamethasone-treatment dramatically reduced the myeloperoxidase activity in the ligated gland (*n* = 5, *P* < 0.05) compared with contralateral control gland levels (Figure 3). Myeloperoxidase was significantly lower in control dexamethasone-treated glands compared with control glands (*P* < 0.01, unpaired Student's *t* test).

Glandular content of secretory peroxidase activity was reduced by 29% after 1 day of duct blockage (743 ± 132 nmol DCF g^−1^*vs* 530 ± 97 nmol DCF g^−1^, *n* = 6) and by 50% (878 ± 86 nmol DCF g^−1^ vs 438 ± 52 nmol DCF g^−1^, *n* = 5, *P* < 0.02) in the dexamethasone-treated ligated group (Figure 1c). Total glandular content of protein was reduced by 27% in dexamethasone-treated ligated glands (635 ± 123 *μ*g g^−1^*vs* 876 ± 107 *μ*g g^−1^, *n* = 7) compared with contralateral control glands, which shows a similar trend to secretory peroxidase. In ligated glands there was a 3% reduction in glandular total protein content (827 ± 79 *μ*g g^−1^*vs* 849 ± 52 *μ*g g^−1^*n* = 6), which was not statistically significant.

### Submandibular secretion

**Figure 4:**
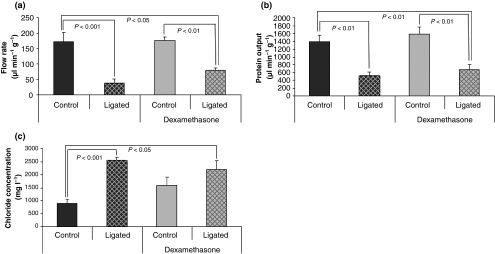
Effects of 24-h duct ligation alone and in the presence of dexamethasone on submandibular gland function. (**a**) The mean flow rate of methacholine-evoked saliva (in units of microlitres per minute per gram gland wet weight) from the 24-h ligated glands was reduced by 78% compared with control glands (*n* = 7). In the dexamethasone-treated ligated glands, flow rate was reduced by 56% compared with contralateral control submandibular glands (*n* = 5) and by 55% compared with control untreated submandibular glands. Following duct ligation, salivary gland ducts were cannulated posterior to the site of clip ligation. (**b**) Salivary protein output induced by isoprenaline with methacholine is significantly reduced in both 24-h duct ligated (*n* = 4) and dexamethasone-treated ligated glands (*n* = 5). (**c**) Mean chloride concentration of methacholine-evoked saliva was increased in the 24-h ligated (*n* = 5) and dexamethasone-treated ligated glands (*n* = 6), when compared with the control untreated glands

The methacholine (12 *μ*g min^−1^ kg^−1^) evoked flow rate from dexamethasone-treated ligated submandibular glands was decreased (*n* = 5, *P* < 0.01) by 56% (79 ± 9 *μ*l min^−1^ g^−1^) compared with contralateral control glands (177 ± 11 *μ*l min^−1^ g^−1^) and by 55% (*P* < 0.05) compared with control untreated glands. In the absence of dexamethasone, the flow rate from ligated glands decreased (*n* = 7, *P* < 0.001) by 78% compared with contralateral glands, (Figure 4a). There was no statistically significant difference between salivary flow rate in ligated glands compared with dexamethasone-treated ligated glands.

Salivary protein output evoked by combined isoprenaline and methacholine was reduced by 58% (*n* = 5, *P* < 0.01) from dexamethasone-treated ligated glands (671 ± 133 *vs* 1583 ± 185 *μ*g min^−1^ g^−1^) and reduced by 63% (*n* = 4, *P* < 0.01) from the ligated glands (518 ± 84 *vs* 1394 ± 151 *μ*g min^−1^ g^−1^) compared with respective contralateral control glands, as seen in Figure 4b. Dexamethasone-treated ligated glands had a lower protein output compared with control untreated glands (*P* < 0.01).

The chloride concentration in saliva from duct ligated glands (2546 ± 123 *vs* 882 ± 170 mg l^−1^, *n* = 5, *P* < 0.001) and dexamethasone-treated ligated glands (*P* < 0.01) was significantly greater than that from control glands. While chloride concentration in saliva from the dexamethasone-treated ligated glands (2202 ± 336 *vs* 1593 ± 310 mg l^−1^, *n* = 6), was not significantly increased (Figure 4c) compared with contralateral control glands.

## Discussion

Dexamethasone treatment reversed salivary gland inflammation and yet the most important markers of salivary gland function, flow rate, protein secretion and chloride concentration indicated that hypofunction persisted. Total salivary protein output, in response to isoprenaline-methacholine stimulation, reveals mainly acinar cell protein secretory function. In our experiments, the reduction in this parameter indicates impairment of the normal secretory process, which has previously been reported as a consequence of duct blockage (Osailan *et al*, 2006a; Carpenter *et al*, 2007). Salivary chloride concentration was analysed as a marker of ductal cell absorptive function. In the dexamethasone-treated ligated glands, chloride concentration was still increased by 150%, compared with the control untreated glands, indicating impaired duct reabsorption. The low salivary flow from these glands would normally result in low salivary levels of chloride, as chloride concentration is correlated with salivary flow rate (Thaysen *et al*, 1954; Martinez *et al*, 1982).

Earlier, we reported the presence of infiltrating inflammatory cells and salivary gland hypofunction, following acute experimental duct ligation, (Carpenter *et al*, 2007). The reduction in salivary secretion in the absence of widespread damage, suggested an influence of inflammatory cells on parenchymal function. The release of cytokines by inflammatory and glandular cells has been suggested to decrease the release of neurotransmitters and the response of the parenchymal cells to neurotransmitters (Fox and Stern, 2002). It has also been shown that exogenous prostaglandins (PGE_1_, PGE_2_ and PG_2*α*_) markedly reduced cholinergic-evoked submandibular gland flow of saliva (Yu, 1986). Experiments using a systemic lipopolysaccharide infusion induced inhibition of salivation, which appeared to be mediated by increased PGE production, via nitric oxide release and cyclooxygenase activation (Lomniczi *et al*, 2001). In Sjögren's syndrome there are increased levels of inflammatory mediators (PGE_2_, thromboxane B2, IL-2 and IL-6) in saliva, which can assist in the diagnosis (Tishler *et al*, 1996, 1997; Streckfus *et al*, 2001; Kaufman and Lamster, 2002). These studies suggest a role of inflammatory mediators in salivary gland hypofunction.

The aim of the present work was to evaluate if the glucocorticoid dexamethasone could preserve salivary function, following duct obstruction. Dexamethasone is an extremely potent and often used anti-inflammatory drug, known to efficiently inhibit polymorphonuclear cell function and tissue invasion, key factors in the host's acute inflammatory response (Perretti *et al*, 2002). In a previous study, it was observed that several administrations of dexamethasone improved salivary secretion after 3 days of a retrograde infusion of adenovirus in the submandibular gland (Adesanya *et al*, 1996). In the duct ligation model of inflammation, together with the inflammatory cell infiltrate, there is also fluid exudate leading to an increased extracellular fluid. It could be hypothesized that this exudate contributed to salivary hypofunction, as it leads to the loss of the normal compact glandular structure. This aspect of the inflammatory reaction was also overcome, as dexamethasone efficiently reduced oedema in the inflamed tissues. To determine the effectiveness of dexamethasone, besides a histological examination of the number of neutrophils in H&E stained slides, myeloperoxidase activity was assayed as an indicator of tissue neutrophil accumulation (Krawisz *et al*, 1984; Thomas *et al*, 1994; Barocelli *et al*, 2006; Xinmin *et al*, 2006). Myeloperoxidase activity was increased after 24 h of duct ligation, correlating to the histological findings, and dramatically reduced after treatment with dexamethasone.

Salivary gland weight was reduced in dexamethasone-treated ligated submandibular glands compared with the contralateral side. Gland histology suggests that parenchymal degranulation together with decreased acinar volume and lack of inflammatory oedema, probably explain the difference in the gland weight. Noticeably, most of the acini appeared to be smaller in the ligated glands compared with control glands. To obtain a direct estimation of the acinar size, further morphometric analyses were undertaken. The mean area showed a reduction in the size of acini in the ligated glands, not only in the dexamethasone-treated, but also in the non-treated group. From earlier studies, it was considered that 24 h after ligation, the degree of atrophy was not marked (Tamarin, 1971; Shiba *et al*, 1972). Our results suggest that the mechanism of acinar atrophy is switched on very early, regardless of the presence of an inflammatory infiltrate. Unlike long-term duct ligation in which glandular shrinkage is due to cell death (Walker and Gobe, 1987; Scott *et al*, 1999), no evident signs of apoptosis were found at 24 h, which agrees with earlier studies (Takahashi *et al*, 2000; Carpenter *et al*, 2007). Consistent with acinar atrophy, tissue levels of peroxidase, a stored acinar cell secretory protein, were reduced in the dexamethasone-treated duct ligated glands. In the untreated group, glandular peroxidase concentration was decreased, but not significantly reduced, as found previously (Osailan *et al*, 2006b). In the dexamethasone-treated group, the significant reduction in peroxidase levels (50% in the dexamethasone-treated ligated gland *vs* 21% in the ligated gland) appears to be consistent with a greater reduction in the acinar size (40% in the dexamethasone-treated ligated gland *vs* 21% in the ligated gland). Total protein from glandular homogenates includes all proteins from different cell populations in the submandibular gland. The reduction of glandular proteins in the dexamethasone-treated ligated gland would support the evidence of an atrophic hypofunctioning gland. In the ligated glands, protein content is similar to control glands. The presence of inflammatory cells in the ligated glands may increase protein concentration and offset any reduction because of acinar size and content of proteins. This result suggests that acute hypofunction of the submandibular gland, in the absence of an inflammatory response, is linked with early atrophy as measured by organ and acinar size, total glandular proteins, stored acinar peroxidase and abrupt reduction in salivary secretion. Histologically, AB/PAS staining showed degranulation of parenchymal cells and enlarged ductal lumina, in both dexamethasone-treated and untreated ligated glands, compared with respective control glands. Apparent early atrophy will further be investigated in the future, as acinar atrophy is generally associated with chronic active inflammation.

It was initially hypothesized that dexamethasone could reverse salivary hypofunction. Some studies have justified its beneficial role in salivary (Adesanya *et al*, 1996) and lung epithelial cells, as dexamethasone is known to inhibit inducible nitric oxide synthase and cyclooxygenase-2 (Robbins *et al*, 1994). The present data indicate that the inflammatory process after 24 h of duct blockage is not the main mechanism accounting for the reduction in salivary acinar secretion. This fact suggests that a non-inflammatory mechanism, possibly early acinar atrophy, leads to salivary gland hypofunction. Additional studies of the gene and protein expression patterns in the ligated submandibular gland may be relevant to determine specific mediators that induce atrophy and salivary hyposecretion. Transforming growth factors-*α* and *β*_1_, platelet-derived growth factor and reactive oxygen intermediates seem to be involved in glandular atrophy. In particular, endogenous IL-10, a crucial regulator of fibrogenetic processes that limits the severity of fibrosis and glandular atrophy will be further studied (Demols *et al*, 2002).

Understanding the mechanisms underlying early salivary hypofunction will help in selecting the best therapeutic option for clinically evident dry mouth and symptomatic xerostomia. To the best of our knowledge, it is the first time that inflammation is abolished from acute hypofunctioning submandibular glands using the duct ligation model. Our data showed that in the absence of inflammation, submandibular gland function is still impaired. A novel finding was the appearance of atrophy only 24 h after the initial insult.

## References

[b1] Adesanya MR, Redman RS, Baum BJ, O’Connell BC (1996). Immediate inflammatory responses to adenovirus-mediated gene transfer in rat salivary glands. Hum Gene Ther.

[b2] Anderson LC, Garrett JR, Zhang X, Proctor GB, Shori DK (1995). Differential secretion of proteins by rat submandibular acini and granular ducts on graded autonomic nerve stimulations. J Physiol Online.

[b3] Barocelli E, Ballabeni V, Ghizzardi P (2006). The selective inhibition of inducible nitric oxide synthase prevents intestinal ischemia-reperfusion injury in mice. Nitric Oxide.

[b4] Beale T, Madani G (2006). Anatomy of the salivary glands. Seminars in Ultrasound, CT, and MRI.

[b5] Carpenter GH, Osailan SM, Correia P, Paterson KP, Proctor GB (2007). Rat salivary gland ligation causes reversible secretory hypofunction. Acta Physiologica.

[b6] Cummins M, Dardick I, Brown D, Burford-Mason A (1994). Obstructive sialadenitis: a rat model. J Otolaryngol.

[b7] Dawson LJ, Fox PC, Smith PM (2006). Sjogrens syndrome – the non-apoptotic model of glandular hypofunction. Rheumatology (Oxford).

[b8] Demols A, Van Laethem JL, Quertinmont E (2002). Endogenous interleukin-10 modulates fibrosis and regeneration in experimental chronic pancreatitis. AJP – Gastrointest Liver Physiol.

[b9] Drage NA, Brown JE, Escudier MP, Wilson RF, Mcgurk M (2002). Balloon dilatation of salivary duct strictures: report on 36 treated glands. Cardiovasc Intervent Radiol.

[b10] Fox RI, Stern M (2002). Sjögren's syndrome: mechanisms of pathogenesis involve interaction of immune and neurosecretory systems. Scand J Rheumatol.

[b11] Giger R, Mhawech P, Marchal F, Lehmann W, Dulguerov P (2005). Mucoepidermoid carcinoma of Stensen's duct: a case report and review of the literature. Head Neck.

[b12] Harrison JD, Epivatianos A, Bhatia SN (1997). Role of microliths in the aetiology of chronic submandibular sialadenitis: a clinicopathological investigation of 154 cases. Histopathology.

[b13] Humphreys-Beher MG, Peck AB (1999). New concepts for the development of autoimmune exocrinopathy derived from studies with the NOD mouse model. Arch Oral Biol.

[b14] Jick H, Pinals R, Ullian R, Slone D, Muench H (1965). Dexamethasone and dexamethasone–aspirin in the treatment of chronic rheumatoid arthritis: a controlled trial. The Lancet.

[b15] Kaufman E, Lamster IB (2002). The diagnostic applications of saliva – a review. Crit Rev Oral Biol Med.

[b16] Kimura-Shimmyo A, Kashiwamura SI, Ueda H (2002). Cytokine-induced injury of the lacrimal and salivary glands. J Immunother.

[b17] Krawisz JE, Sharon P, Stenson F (1984). Quantitative assay for acute intestinal inflammation based on myeloperoxidase activity. Gastroenterology.

[b18] Lomniczi A, Mohn C, Faletti A (2001). Inhibition of salivary secretion by lipopolysaccharide: possible role of prostaglandins. AJP – Endocrinol Metab.

[b19] Lustmann J, Regev E, Melamed Y (1990). Sialolithiasis: a survey on 245 patients and a review of the literature. Int J Oral Maxillofac Surg.

[b20] Mandel ID, Baurmash H (1980). Sialochemistry in chronic recurrent parotitis – electrolytes and glucose. J Oral Pathol Med.

[b21] Martinez JR, Bylund DB, Cassity N (1982). Progressive secretory dysfunction in the rat submandibular gland after excretory duct ligation. Arch Oral Biol.

[b22] Masferrer JL, Zweifel BS, Seibert K, Needleman P (1990). Selective regulation of cellular cyclooxygenase by dexamethasone and endotoxin in mice. J Clin Invest.

[b23] Norberg LE, Abok K, Lundquist PG (1988). Effects of ligation and irradiation on the submaxillary glands in rats. Acta Otolaryngol (Stockh).

[b24] Ohno K, Hattori T, Kagami H, Ueda M (2007). Effects of preceding sialadenitis on the development of autoimmunity against salivary gland. Oral Dis.

[b25] Osailan SM, Proctor GB, Carpenter GH, Paterson KL, Mcgurk M (2006a). Recovery of rat submandibular salivary gland function following removal of obstruction: a sialometrical and sialochemical study. Int J Exp Pathol.

[b26] Osailan SM, Proctor GB, Mcgurk M, Paterson KL (2006b). Intraoral duct ligation without inclusion of the parasympathetic nerve supply induces rat submandibular gland atrophy. Int J Exp Pathol.

[b27] Perretti M, Chiang N, La M (2002). Endogenous lipid- and peptide-derived anti-inflammatory pathways generated with glucocorticoid and aspirin treatment activate the lipoxin A4 receptor. Nat Med.

[b28] Proctor GB, Chan KM (1994). A fluorometric assay of peroxidase activity utilizing 2′,7′-dichlorofluorescein with thiocyanate: application to the study of salivary secretion. J Biochem Biophys Methods.

[b29] Proctor GB, Carpenter GH, Segawa A, Garrett JR, Ebersole L (2003a). Constitutive secretion of immunoglobulin A and other proteins into lumina of unstimulated submandibular glands in anesthetised rats. Exp Physiol.

[b30] Proctor GB, Garrett JR, Carpenter GH, Ebersole LE (2003b). Salivary secretion of immunoglobulin A by submandibular glands in response to autonomimetic infusions in anaesthetised rats. J Neuroimmunol.

[b31] Qi S, Liu X, Wang S (2005). Sialoendoscopic and irrigation findings in chronic obstructive parotitis. Sialoendoscopy; irrigation fluid; sialography; chronic obstructive parotitis. Laryngoscope.

[b32] Rettori V, Lomniczi A, Elverdin JC (2000). Control of salivary secretion by nitric oxide and its role in neuroimmunomodulation. Ann NY Acad Sci.

[b33] Robbins RA, Springall DR, Warren JB (1994). Inducible nitric oxide synthase is increased in murine lung epithelial cells by cytokine stimulation. Biochem Biophys Res Commun.

[b34] Rosignoli F, Goren NB, Perez Leiros C (2001). Alterations in nitric oxide synthase activity and expression in submandibular glands of NOD mice. Clin Immunol.

[b35] Scott J, Liu P, Smith PM (1999). Morphological and functional characteristics of acinar atrophy and recovery in the duct-ligated parotid gland of the rat. J Dent Res.

[b36] Shiba R, Hamada T, Kawakatsu K (1972). Histochemical and electron microscopical studies on the effect of duct ligation of rat salivary glands. Arch Oral Biol.

[b37] Shori DK, Proctor GB, Garrett JR, Zhang XS, Carpenter GH (1997). Histochemical staining of ducts in submandibular glands by DMAB-nitrite detects stored tissue kallikreins. Biochem Soc Trans.

[b38] Standish SM, Shafer WG (1957). Serial histologic effects of rat submaxillary and sublingual salivary gland duct and blood vessel ligation. J Dent Res.

[b39] Streckfus C, Bigler L, Navazesh M, Al-Hashimi I (2001). Cytokine concentrations in stimulated whole saliva among patients with primary Sjögren's syndrome, secondary Sjögren's syndrome, and patients with primary Sjögren's syndrome receiving varying doses of interferon for symptomatic treatment of the condition: a preliminary study. Clin Oral Investig.

[b40] Takahashi S, Nakamura S, Suzuki R (2000). Apoptosis and mitosis of parenchymal cells in the duct-ligated rat submandibular gland. Tissue Cell.

[b41] Tamarin A (1971). Submaxillary gland recovery from obstruction. I. Overall changes and electron microscopic alterations of granular duct cells. J Ultrastruct Res.

[b42] Tamarin A (1979). The leukocytic response in ligated rat submandibular glands. J Pathol.

[b43] Tanda N, Ohyama H, Yamakawa M (1998). IL-1beta and IL-6 in mouse parotid acinar cells: characterization of synthesis, storage, and release. AJP – Gastrointest Liver Physiol.

[b44] Thaysen JH, Thorn NA, Schwartz IL (1954). Excretion of sodium, potassium, chloride and carbon dioxide in human parotid saliva. AJP – Legacy.

[b45] Thomas EL, Jefferson MM, Joyner RE, Cook GS, King CC (1994). Leukocyte myeloperoxidase and salivary lactoperoxidase: identification and quantitation in human mixed saliva. J Dent Res.

[b46] Tishler M, Yaron I, Raz A, Meyer FA, Yaron M (1996). Salivary eicosanoid concentration in patients with Sjögren's syndrome. Ann Rheum Dis.

[b47] Tishler M, Yaron I, Shirazi I, Yaron M (1997). Saliva: an additional diagnostic tool in Sjogren's syndrome. Semin Arthritis Rheum.

[b48] Van Den Akker HP, Busemann-Sokole E (1983). Submandibular gland function following transoral sialolithectomy. Oral Surg Oral Med Oral Pathol.

[b49] Walker NI, Gobe GC (1987). Cell death and cell proliferation during atrophy of the rat parotid gland induced by duct obstruction. J Pathol.

[b50] Wang SL, Zou ZJ, Wu QG, Sun KH (1992). Sialographic changes related to clinical and pathologic findings in chronic obstructive parotitis. Int J Oral Maxillofac Surg.

[b51] Xinmin D, Yunyou D, Chaosheng P (2006). Dexamethasone treatment attenuates early seawater instillation-induced acute lung injury in rabbits. Pharmacol Res.

[b52] Yamakawa M, Weinstein R, Tsuji T, Mcbride J, Wong DTW, Login GR (2000). Age-related alterations in IL-1beta, TNF-alpha, and IL-6 concentrations in parotid acinar cells from BALB/c and non-obese diabetic mice. J Histochem Cytochem.

[b53] Yang YH, Hutchinson P, Santos LL, Morand EF (1998). Glucocorticoid inhibition of adjuvant arthritis synovial macrophage nitric oxide production: role of lipocortin 1. Clin Exp Immunol.

[b54] Yu JH (1986). Modulating effects of prostaglandins on parasympathetic-mediated secretory activities of rat salivary glands. Prostaglandins.

